# Preventing Sexual Harm in Nightlife Settings: A Scoping Review

**DOI:** 10.1007/s10508-024-02872-y

**Published:** 2024-05-09

**Authors:** Kira Button, Nicholas Taylor, Tahnee Guala, Dominique De Andrade, Kerri Coomber, Zara Quigg, Peter Miller

**Affiliations:** 1 School of Psychology, Deakin University, Geelong Waterfront Campus, 1 Gheringhap Street, Geelong, VIC 3220 Australia; 2 National Drug Research Institute, Curtin University, Melbourne, Australia; 3 Burnet Institute, Melbourne, Australia; 4 Griffith Criminology Institute, Griffith University, Brisbane, Australia; 5 School of Psychology, University of Queensland, Brisbane, Australia; 6 Public Health Institute, Liverpool John Moores University, Liverpool, UK

**Keywords:** Sexual harm, Nightlife, Bystander interventions, Prevention, Alcohol

## Abstract

Sexual harm within nightlife settings is a pervasive global concern; however, little is known about the nature of available interventions. The current study aims to review the literature on the nature and effectiveness of nightlife-related sexual harm interventions. A systematic literature search of six databases was conducted to identify records that were published between 1970 and June 2023 and reported approaches that aimed to reduce or prevent nightlife-related sexual harm. Records were included if they theorized, discussed, or evaluated an intervention, prevention or response strategy; however, individual safety strategies were excluded. Results were categorized according to intervention type. Thirty-five peer-reviewed journal articles and 16 gray literature records were identified. The most common nightlife-related sexual harm intervention strategies covered by the literature included targeted policies, laws, and regulations, bystander interventions, and awareness-raising campaigns. Literature in the area is increasing, with the majority of the articles (77.1%) being published in the previous six years; however, there are very few interventions that have been critically evaluated (22.9%). Promising areas for intervention include targeted alcohol regulations (e.g., lockout policies), venue-level policies, and environmental interventions. However, an increase in rigorous evaluative practices is urgently required to ensure future interventions are based on sound theoretical work and empirical evidence.

## Introduction

Sexual harm is a term that encompasses a broad spectrum of unwanted sexual conduct and behaviors, often described in existing literature as “sexual harassment,” “sexual violence,” “sexual aggression,” or “unwanted sexual attention” (Fileborn, 2016; Mellgren et al., 2018; Quigg et al., 2020; Sanchez et al., 2019). There is a growing body of evidence documenting high rates of sexual harm in nightlife settings internationally, with lifetime prevalence rates being greater than 50% for females across numerous studies utilizing in-depth interviews and focus groups (Becker & Tinkler, 2015; Gunby et al., 2017; Huber & Herold, 2006; Kavanaugh, 2013). Nightlife settings are characterized by the presence of entertainment venues such as pubs, clubs, and bars within a relatively small geographic area (Brunn et al., 2021). Research indicates that males experience nightlife-related sexual harm substantially less than their female counterparts (Becker & Tinkler, 2015; Johnson et al., 2015; Tinkler et al., 2018) and are also more likely to perpetrate such harm (Fung et al., 2021; Santos et al., 2015). While nightlife venues play an important role in the social development of young adults by providing a unique setting for individuals to socialize, unwind, and have fun (Aresi & Pedersen, 2016; Hadfield, 2019), evidence suggests that a multitude of individual and environmental factors contribute to and exacerbate the risk of sexual harm within these settings (Quigg et al., 2020).

Risky drinking behaviors (e.g., pre-drinking and binge drinking) are common among nightlife patrons, with research indicating that alcohol consumption is positively associated with sexual harm victimization and perpetration (e.g., Fung et al., 2021; Santos et al., 2015). Other factors implicated in nightlife-related sexual harm include venue-level crowding, venue type, and sociocultural norms (Kavanaugh & Anderson, 2009; Sanchez et al., 2019; Thompson & Cracco, 2008). It has been suggested that the highly sexualized nature of some nightlife venues may facilitate and encourage sexual harm within these contexts (Fileborn, 2012). Despite concerning prevalence rates of sexual harm (estimated at 20–60%; Baldwin et al., 2022a; Gunby et al., 2017; Quigg et al., 2020), research pertaining to the prevention and response to sexual harm in this context is limited, with little known about the breadth and effectiveness of existing interventions (Quigg et al., 2020).

Early prevention efforts focused on individual responsibility and encouraged patrons, particularly women, to take precautions themselves to prevent sexual harm (Richards, 1991; Schwartz et al., 2000). Richards (1991) recommended that prevention measures should include social skills training to ensure women are aware of their outward demeanor and teach them to control the nonverbal messages that they send to men. It was suggested that such training may empower women to become aware and reduce their risk of being victimized (Parks et al., 2008). Similarly, in the late nineties following the increased prevalence of drug-facilitated sexual assaults in club and rave scenes, patrons were encouraged to take precautions to prevent their drinks from being spiked (e.g., “don’t leave drinks unattended”; Payne-James & Rogers, 2002; Schwartz et al., 2000). More recent focus group and in-depth interview research indicates that patrons continue to engage in individual preventative measures to avoid sexual harm and keep safe within nightlife settings (Anitha et al., 2021; Brooks, 2011; Gunby et al., 2020). Strategies frequently reported by patrons include reducing or monitoring alcohol consumption, covering the top of their drink, and taking their drink to the bathroom with them (Brooks, 2011; Graham et al., 2017). Other safety tactics adopted by women were not dressing provocatively, avoiding particular venues, staying with friends, and pretending to have a partner (Brooks, 2011; Graham et al., 2017; Gunby et al., 2020). Patrons recognize the need to take responsibility for their own safety, with these safety precautions being described as a necessity on a night out (Brooks, 2011).

Recent social justice movements such as “Me Too” and “Times Up” have helped shift the traditional victim-responsibility discourse of sexual harm (Wexler et al., 2019; Williams et al., 2019). Such feminist movements were responsible for highlighting the magnitude and severity of sexual violence among women, with the “Me Too” hashtag being used over 12 million times in the first 24 h of its creation (CBS, 2017). While individual safety strategies remain a common practice, prevention tips that focus on changing aspects of victim behavior may reinforce the notion that victims are, in some way, responsible for being victimized (Cherniawsky & Morrison, 2022). A recent novel study utilizing a between-participants experimental method found that participants who received victim-focused prevention strategies (e.g., “don’t leave drinks unattended”) attributed more blame to victims of sexual harm in a vignette compared to participants who received perpetrator-focused prevention tips (e.g., “don’t assume someone’s choice of clothing means they want to have sex with you”; Cherniawsky & Morrison, 2022). It should be noted that while the generalizability of these findings may be limited due to the sample being predominantly White undergraduate students, it is anticipated that the negative effects of victim-focused prevention tips would be amplified in samples with lower educational attainment (Cherniawsky & Morrison, 2022; Vonderhaar & Carmody, 2015).

Cherniawsky and Morrison (2022) suggest that certain theoretical perspectives, such as the rape myth acceptance theory provide a foundational understanding of victim-blaming within the context of victim-focused prevention strategies. It should be noted that, to date, theoretical frameworks have rarely been used to inform any type of sexual harm prevention strategy within the nightlife context. The rape myth acceptance theory is grounded in traditional gender norms and posits that misconceptions regarding sexual harm often circulate in society (e.g., through media and social groups), which can cause individuals to misattribute blame in real sexual harm situations (Burt, 1980; Payne et al., 1999). Widespread beliefs that direct blame toward the victim include condemning victims for drinking alcohol, dressing “inappropriately,” or acting seductively (Parks et al., 1998; Rape Crisis England & Wales, 2022). Common beliefs that act to exonerate the perpetrator include “women often lie about being raped” and “men can’t help themselves once they’re turned on” (Rape Crisis England & Wales, 2022). Rape myth acceptance perpetuates sexual harm by simultaneously placing blame on the victim, excusing the perpetrator, and justifying or minimizing the harm (Payne et al., 1999). Researchers have demonstrated that nightlife-related bystander intervention programs can reduce individuals’ rape myth acceptance (Powers & Leili, 2018; Quigg et al., 2021). Further, it has been proposed that bystander interventions and awareness-raising campaigns may alleviate the responsibility of the recipient of sexual harm by placing the accountability on members of the community instead (i.e., other patrons and venue staff; Powers & Leili, 2016; Quigg et al., 2021). However, a review of the literature regarding nightlife-related sexual violence (published up until 2018) found that there had been very few empirically tested bystander interventions or awareness campaigns implemented in nightlife venues (Quigg et al., 2020).

Little is known about the nature and effectiveness of preventative measures which aim to reduce sexual harm in nightlife settings. In order to develop and implement evidence-based interventions to address sexual harm, a thorough understanding of existing strategies is imperative. Quigg et al. (2020) conducted a recent scoping review investigating the nature, prevalence, associated factors, and prevention of nightlife-related sexual violence. The current study will build on this research by utilizing a broader search strategy exclusively relating to sexual harm prevention and conducting an additional gray literature search. Additionally, as noted by Quigg et al. (2020), this is a growing area of research; therefore, it is expected that a number of additional articles will have been published since 2018. The aim of the current study is to develop a comprehensive understanding of preventative responses to nightlife-related sexual harm. To fulfill this objective, this review will answer the following research question: What intervention or prevention strategies have been theorized, developed, or implemented internationally to reduce or prevent sexual harm in nightlife settings? The current paper will not include studies that discuss individual safety strategies, as victim-focused approaches may perpetuate victim-blaming (Cherniawsky & Morrison, 2022).

## Method

### Search Strategy

A systematic literature search was undertaken to identify articles that described any intervention or prevention strategy that has been implemented to prevent the occurrence of sexual harm within and around nightlife settings (i.e., pubs, clubs, and bars). Peer-reviewed literature searches were undertaken in EMBASE, Scopus, Medline Complete, CINAHL Complete, APA PsycINFO, and APA PsycArticles on June 16, 2023. A search strategy was developed using a combination of terms for “sexual harm,” “nightlife,” and “interventions” (Appendix). The search strategy was adapted for each database, and filters were used to only include sources published in English. The reference lists were screened using forward and backward snowballing to identify additional studies. To develop a complete overview of existing interventions, a gray literature search was conducted on June 13, 2023, guided by existing systematic search methods (Godin et al., 2015; Mahood et al., 2014). The search strategy was used to search the first 10 pages of Google (first 100 websites) and Google Scholar (first 100 records). The current scoping review has been developed in accordance with the Joana Briggs Institute (JBI) methodological guidelines (Peters et al., 2021), and a protocol was developed in accordance with the JBI model (https://osf.io/68ye5).

### Inclusion and Exclusion Criteria

Articles were included if they contained any discussion of strategies that aimed to reduce, prevent, or respond to sexual harm within nightlife settings. Qualitative, quantitative, and mixed-methods designs and reviews were included. Letters to the editor, editorials, commentaries, and conference abstracts were excluded. The articles were not included if they exclusively discussed individual safety strategies (e.g., not dressing provocatively), as these interventions solely relied on personal responsibility.

### Study Selection

**Fig. 1:**
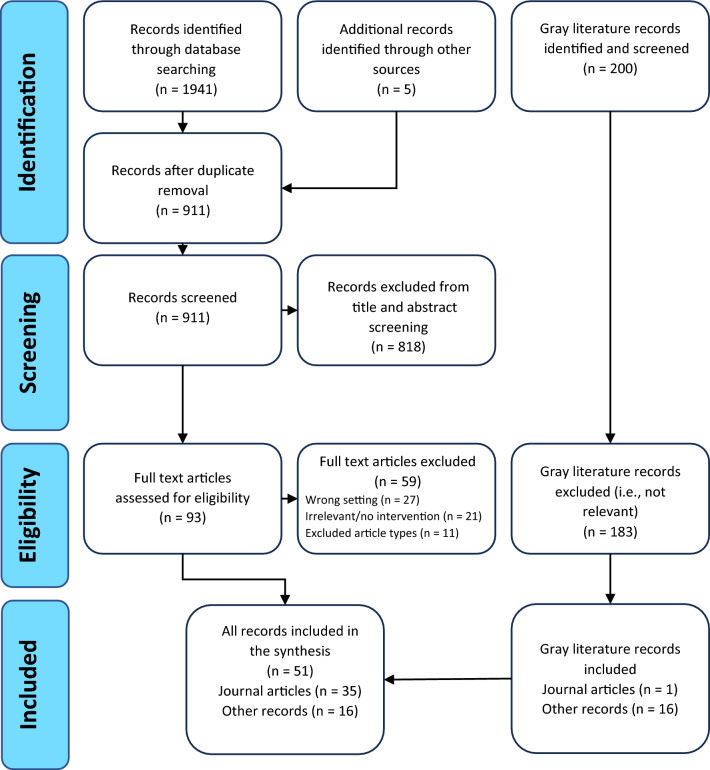
PRISMA flow diagram (Moher et al., 2009)

The database searches yielded 1941 potentially relevant articles (Fig. 1). Following duplicate removal, 911 titles and abstracts were screened by the lead author (KB). As per established methods, a second reviewer (TG) screened a randomly selected 10% of articles (Baldwin et al., 2022b); 3% of this subset had discrepant coding, and disagreements were resolved through discussion. Covidence was used to screen all articles (Covidence Systematic Review Software, 2022). The full text of 93 articles was screened by the lead author (KB; with a second reviewer [TG] screening a randomly selected 10% to ensure consistency), and 29 were included in the review. A further 5 journal articles were identified through reference list searching, and 1 peer-reviewed article was identified in the gray literature search, resulting in 35 journal articles being included in the review. The gray literature search yielded an additional 16 records (14 from Google and 2 from Google Scholar).

### Data Extraction and Analysis

All data were extracted by the lead author (KB) and verified by the second reviewer (TG). Information that was extracted included year of publication, country, methodology, and data type (i.e., quantitative, qualitative, or mixed methods). The type of sexual harm evaluated or discussed in the paper was also extracted. It is important to highlight the type of sexual harm being targeted, as a strategy effective for reducing unwanted sexual attention may not be effective for addressing sexual assault. The sample characteristics were described where applicable (e.g., gender, age range, and mean). The studies were categorized by intervention type, with a brief description of the intervention noted. The typology was originally developed from previous work by Quigg et al. (2020) and was adjusted in response to the records that emerged. The categories were not mutually exclusive, with some studies discussing more than one intervention type. If studies were evaluated, data on outcome measures were also recorded. If studies were not evaluated, the relevant themes and findings were noted. Data from the gray literature records were included in a separate table. A qualitative synthesis of existing nightlife-related sexual harm prevention and response strategies is presented.

## Results

### Characteristics of Included Peer-Reviewed and Gray Literature

Thirty-five peer-reviewed journal articles described a nightlife-related sexual harm intervention or prevention strategy. The majority of studies were conducted in the UK (37.1%), followed by the USA (22.9%), Canada (8.6%), Australia (11.4%), Spain (8.6%), and India (8.6%). Three (8.6%) studies utilized data from several countries. A large proportion of the studies identified (77.1%) were published in the past 6 years (2017–2023). Of the studies, 18 (51.4%) were qualitative, 15 (42.9%) were quantitative, and 2 (5.7%) utilized mixed methods. Four (11.4%) of the 35 studies included venue staff as participants**.** Eight (22.9%) studies evaluated the effectiveness of an intervention strategy or policy in reducing sexual harm or related factors (e.g., staff members readiness to intervene). Table 1 characterizes the articles by intervention type and provides a description of the relevant content of the articles. The majority of studies investigated policies, laws, and regulations (22.9%) and bystander-related interventions (20%), followed by awareness-raising campaigns (8.6%) and the implementation of trained care worker teams (2.9%). Nineteen (54.3%) studies discussed some form of prevention strategy more generally (i.e., not a specific intervention). Table 2 provides a summary of each study that was included in the review.

**Table 1:** Categorizing peer-reviewed studies by intervention type

Intervention	Description	Citations
Policies, Laws, and Regulations	Venue level (e.g., safe space policies) and state/district level (e.g., alcohol policies) policies or regulations that were implemented to directly or indirectly reduce sexual harm	Benny et al. (2019); De Vocht et al. (2016); Hill et al. (2020); Hill and Megson (2020); Khurana and Mahajan (2022); Lippy and DeGue (2016); Palk et al. (2010); Toomey et al. (2012)
Bystander-related Interventions	i. Venue staff intervention (e.g., venue staff bystander training) ii. Patron intervention	i. Graham et al. (2014); Hill and Megson (2020); Powers and Leili (2018); Quigg et al. (2021); Quigg et al. (2022) ii. Graham et al. (2014); Ham et al. (2019); Ham et al. (2022)
Awareness-Raising Campaigns	Exploring the effectiveness of educational/awareness campaigns which aim to reduce or prevent nightlife-related sexual harm	Carline et al. (2018); Gunby et al. (2017); Wood and Shukla (2017)
Implementation of Trained Care Worker Teams	Exploring the effectiveness of groups who are trained to provide practical support to patrons at risk of sexual harm within venues	Garius et al. (2020)
General Discussion	Articles that contained a broad discussion of an intervention. Topics included awareness and safety advice campaigns^a^, bystander strategies^b^, education^c^, venue staff/security^d^, mutual partnerships^e^, environmental interventions^f^, policy^g.^ and barriers and facilitators of prevention^h^	Anitha et al. (2021)^bd^; Brooks (2011)^ab^; Duncan et al. (2022)^g^; Fileborn (2016)^bcg^; Forsyth (2009)^f^; García-Carpintero et al. (2022)^bd^; Gómez et al. (2022)^b^; Green (2022)^d^; Graham et al. (2017)^b^; Gunby et al. (2020)^b^; Hill et al. (2020)^a^; Hill and Megson (2020)^h^; Kavanaugh and Anderson (2009)^bd^; Leone et al. (2022)^b^; Levine (2018)^aeh^; Powers and Leili (2016)^dfh^; Prego-Meliro et al. (2022)^a^; Quigg et al. (2020); Wrightson-Hester and Allan (2022a)^bh^

^a^General discussion topics included awareness and safety advice campaigns

^b^Bystander strategies

^c^Education

^d^Venue staff/security

^e^Mutual partnerships

^f^Environmental interventions

^g^policy

^h^barriers and facilitators of prevention

**Table 2:** Summary of each peer-reviewed study that was included in the current paper

Citation	Country	Data Type	Methodology/Participants	Intervention Type	Sexual Harm Targeted or Mentioned	Description	Key findings and Outcomes
Anitha et al. (2021)	England	Quantitative	Semi-Structured Interviews N = 26 college students 73.1% Female, aged 18—25	General Discussion (venue security, bystander intervention—friends)	Sexual Violence	Discussions around vignettes based on gender-based violence in nightlife venues. Participants discussed possible responses	Some participants felt venue security did not respond appropriately (or at all) to incidents of sexual harm. A strategy used by participants to prevent sexual harm was having male friend/boyfriend present. Male participants reported protecting female friends
Benny et al. (2019)	Canada	Quantitative	Regression-discontinuity approach Police records	Policy	Sexual Assault	Impact of minimum legal drinking age (MLDA) laws on violent victimization (using police reported violent events) (Note: sexual assault was grouped with physical assault, homicide and robbery)	In comparison to individuals slightly younger than the MLDA, females and males just older (of legal drinking age) experienced increases in violent victimization by 44.9% (*p* < .001) and 18.3% (*p* = .001), respectively, in bars/restaurants/open air settings
Brooks (2011)	Scotland	Qualitative	Focus groups (n = 4) and in-depth interviews (n = 29) N = 35 women Aged 18—25	General Discussion (safety advice campaign and bystander intervention)	Drug-assisted Sexual Assault Sexual Violence	Examined young female’s experiences and responses to nightlife-related safety advice. Participants were asked if they were aware of any safety advice campaigns	Recall of specific campaigns were low, although there were high levels of awareness of general key messages (e.g., how to prevent drink spiking, limit alcohol consumption and look out for friends) Strategies used by participants included seeking protection from male friends and seeking assistance from venue staff and security
Carline et al. (2018)	England	Qualitative	Focus Groups (n = 6) N = 41 Males (Aged 18–24)	Awareness-Raising Campaign	Sexual Offending Sexual Violence	Discussing the “Can’t answer? Can’t consent—sex without consent is rape” male-targeted campaign. Described ‘threat campaigns’	Participants understood what the campaign message was, however, there was uncertainty around the meaning of consent. Some participants felt the campaign was too accusing, harsh and sexist. Participants often tried to reduce male responsibility (e.g., she shouldn’t put herself in that situation)
De Vocht et al. (2016)	England	Quantitative	Crime rates were analyzed using hierarchical (log-rate) growth modeling Evaluation	Policy	Sexual Crimes	Using reported crime data to calculate the rates of reported crime (e.g., alcohol-attributable sexual offenses). Regions were then classified based on their cumulative licensing policy intensity	Rates of alcohol-related sexual crimes declined significantly faster in areas with more intense alcohol policies from 2009 to 2013. However, crime rate increased again following this period
Duncan et al. (2022)	Australia	Qualitative	In-depth Interviews N = 15 quantitative alcohol and violence researchers	General Discussion (policy)	Sexual Assault	Interviews explored issues around the relationship between gender, violence, alcohol and related policies	Some participants argued that despite it being men that are most often involved in nightlife-related violence, alcohol-policies may unfairly affect women or others who do not engage in violence. Others argued that the policies are justified as they likely reduce women’s vulnerability to sexual assault. The authors suggest that population-wide alcohol policies may leave men’s anti-social behavior unmarked
Fileborn (2016)	Australia	Qualitative	Focus Groups (N = 16 participants, 62.5% female), In-depth Interviews (N = 4, 100% female) and Online Surveys (N = 252, 61.5% female)	General Discussion (bystander intervention, education, policy)	Unwanted Sexual Attention	Participants were asked to provide suggestions on how to prevent nightlife-related sexual harm	Suggestions included bystander response, prevention through education (e.g., teach women to be better sexual communicators and be more assertive) and increase venue responsibility (e.g., clear policies for staff to respond to unwanted sexual attention)
Forsyth (2009)	Scotland	Mixed Methods	Fieldwork observations N = 8 nightclub observations	General Discussion (environmental intervention)	Sexual Competition	Observers examined how music could impact and control the nightlife experience, including level of disorder and sexual activity among the crowd	Music policy impacted the level of disorder and sexual activity of patrons Music and the DJ were effectively used as a form of “soft policing” to control the crowd
García-Carpintero et al. (2022)	Spain	Qualitative	In-depth Interviews N = 24, 58.3% Male, age (*M* = 17.7), Focus Groups N = 49 (across 6 groups), 75.5% female, aged 18–22 (*M* = 19.5)	General Discussion (bystander intervention, security)	Sexual Violence	One thematic block that the questions were based upon was “experiences of fear in recreational nightlife spaces and strategies put into place to combat these situations”	Female participants reported accompanying other females to risky places (e.g., club bathroom) and having their friends intervene when unwanted sexual attention occurred. They also utilized a “male guardian” who could pretend to be their boyfriend to deter harassment from other males. Participants also reported getting venue security involved in incidents
Garius et al. (2020)	England and Wales	Quantitative	Exploratory analysis of police recorded crime data Evaluation	Implementation of Trained Care Worker Teams	Sexual Crimes	Evaluating the “Drinkaware Crew” (workers provide practical support to patrons at risk of sexual harm in venues). Compared incidents of violent/sexual crime before and after the intervention was implemented in test venues in 2 cities	In City A results were inconclusive. In City B there was an increase in crime within test venues following the intervention. The authors concluded the data used are unreliable for the purpose of evaluation
Gómez et al. (2022)	Spain	Qualitative	In-depth Interview N = 26, 57% Male, aged 16–22 (*M* = 17.7)	General discussion (bystander intervention)	Sexual Violence	One of the thematic segments interview questions were based upon was the prevention and intervention of nightlife-related sexual violence	Female participants reported frequently protecting other females from sexual assault. Some participants also reported that they would appeal for help from nightclub staff
Graham et al. (2014)	Canada	Qualitative	Venue Observations N = 258 observed incidents	Bystander Intervention	Sexual Aggression	Researchers observed sexually aggressive incidents, with one aim being to examine intervention by staff and other patrons	Ten incidents involved staff, with the perpetrator being evicted in only 1 incident. In 24 incidents, friends of the target were involved (in which they intervened in almost every case). Of the 8 incidents involving friends of the perpetrator, 5 involved the friends encouraging or supporting the perpetrator
Graham et al. (2017)	Canada	Quantitative	Online Survey N = 153 females, aged 19 -29 (*M* = 21.8, *SD* = 2.5)	General Discussion (bystander intervention)	Sexual Harassment (unwanted touching and persistent harassment)	Participants were asked what strategies they used to refute sexual harassment	Friends intervened in 93% of incidents of persistence (i.e., a person continuously hitting on you despite indicating you are not interested) and 84% of incidents of unwanted touching
Green (2022)	England	Qualitative	Ethnographic Venue Observation N = 1 venue Semi-structured Interviews N = 15 venue workers, 53.3% female	General Discussion (staff intervention)	Unwanted Sexual Attention	The researcher conducted observations at one bar (of which they were a former employee). One thematic block that interview questions were based on was “strategies or mechanisms venue workers implement while working” (to respond to unwanted sexual advance)	More experienced staff encourage newer staff to report incidents of unwanted sexual attention to management immediately (as new staff often feel as though they must accept this attention)
Gunby et al. (2017)	England	Qualitative	Focus Groups (n = 6) N = 41 Males (Aged 18–24)	Awareness-Raising Campaign	Sexual Violence	Assessing the “Can’t answer? Can’t consent—sex without consent is rape” campaign. Recall and perception of campaign were assessed	Very few participants were aware of the campaign. Participants discussed reasons why it may have been ineffective (e.g., they would not take in information when drunk, campaign signage would be more noticeable if model was prettier/showed more skin)
Gunby et al. (2020)	England	Qualitative	Focus Groups (n = 4) N = 31 Female law students (Aged 18—24)	General Discussion (bystander intervention—friends/boyfriend)	Unwanted Sexual Attention	Participants were asked about their experiences of sexual harm within nightlife venues. Discussions centered on strategies for managing risk	Common themes for managing risk included using men as a protector (e.g., presence of male friend or boyfriend reduced the likelihood of sexual harm) and female camaraderie (e.g., remain vigilant for vulnerable others)
Ham et al. (2019)	USA	Quantitative	Experimental design N = 128, 50% Females, aged 21–29 (M = 23.27, SD = 2.41) Participants were randomly assigned to intoxication (target BAC = 0.08%; n = 64) or sober control group (n = 64)	Bystander Intervention	Alcohol-related Sexual Violence	Assessing the role of bystander intoxication and likelihood of intervening in a sexual assault situation in nightlife setting (using vignettes in a laboratory setting)	The control (sober) group reported greater risk and need to intervene compared to the intoxicated group (*p* < .001) The intoxication and control group did not differ on taking responsibility to act, deciding how to act and acting to intervene
Ham et al. (2022)	USA	Quantitative	Field-based design N = 315 nightlife patrons, 53% Males, aged 21–29 (*M* = 23.17, *SD* = 2.28)	Bystander Intervention	Alcohol-related Sexual Assault	Assessing the role of bystander intoxication and likelihood of intervening in a sexual assault situation in nightlife setting (using vignettes and patron BrAC)	Increased breath alcohol concentration (BrAC) was negatively associated with identifying how uncomfortable the victim was in the vignette (*p* < .01). BrAC was not significantly associated with ratings of dangerousness, confidence to intervene and personal responsibility (*p* > .05). There was a negative indirect association of intoxication on perceived responsibility and confidence to intervene mediated through reduced risk appraisal
Hill et al. (2020)	England	Qualitative	Ethnographic Observations at venues with gigs Interviews with concert goers (n = 7, Aged 21—43), venue promoters (n = 3, 66% female), managers (n = 3, 100% Men)	Policy General Discussion (awareness raising/ advertisement)	Sexual Violence	Discussion of measures used by venues to prevent sexual harm at gig and suggestions from concert goers Observation of a venue with a Safe Space Policy	Concert goers suggested safe space policies. Observer noted that these policies likely act as a preventive to sexual harm The authors suggest a dual approach to prevention; firstly a culture change (e.g., perpetrator education), and an approach that deals with sexual violence when it occurs
Hill and Megson (2020)	UK	Qualitative	Interviews with venue managers (n = 4) and promoters (n = 2)	Bystander Intervention; (e.g., Good-night Out Training [GNO]) Policy General Discussion (barriers and facilitators of prevention)	Sexual Violence	Aimed to understand how and why venues implement sexual violence prevention strategies at gigs, and what are the facilitators and barriers to change	The main barrier for accessing staff training were financial constraints Facilitators of change included networking between venues (i.e., share ideas) and having female owners and promoters GNO bystander component was viewed favorably by managers and promoters
Kavanaugh and Anderson (2009)	USA	Qualitative	In-depth Interviews N = 51, 51% Males, aged 18—32 (*M* = 25.7) Venue Observations N = 29	General Discussion (bystander intervention—friends, venue security)	Sexual Assault	Participants were asked open ended questions about their involvement in nightlife-venues and their experiences of victimization (physical and sexual)	Peer-centered protective strategies were commonly utilized by females (e.g., putting physical space between friend and perpetrator) Utilizing venue security was viewed by females (and not males) as valuable risk management approach for retaliating against perpetrators
Khurana and Mahajan (2022)	India	Quantitative	Natural Experiment (difference-in-difference strategy) Evaluation	Policy	Sexual Assault	Compared changes in reported incidents of sexual assault following a ban on the sale hard-liquor in bars in one state in India (using neighboring states as controls)	The most conservative estimates showed that the liquor ban reduced reported sexual assaults by 10%
Leone et al. (2022)	USA	Quantitative	Online Survey N = 290 undergraduate students, 50% female, age (*M* = 20)	General Discussion (bystander intervention)	Sexual Aggression	Participants completed measures about past bar and club attendance, bystander efficacy and barriers to intervening	Participants who were more confident in their bystander skills were less likely to think it was not their responsibility to intervene in sexual assault incidents (P < .05). Those who frequent bars and clubs more often and had higher bystander efficacy were less likely to be hesitant to intervene due to embarrassment compared to those low in bystander efficacy
Levine (2018)	USA	Qualitative	In-Depth Interviews N = 30 Scientists and Scholars	General Discussion (mutual partnerships, awareness raising and prevention, barriers and facilitators of prevention)	Sexual Violence	Participants were asked about factors that enable and constrain nightlife-related sexual harm research	Promising prevention strategies discussed included cultivating mutually beneficial partnerships with venues and combining awareness raising and prevention. Barriers to intervention discussed included resource constraints, venue finances and building relationships with venues
Lippy and DeGue (2016)	International	Qualitative	Literature Review	Policy	Sexual Violence	Investigating existing evidence regarding the effects of several alcohol policy’s on sexual violence perpetration	The majority of studies indicated that higher alcohol prices and taxes were associated with lower rates of sexual violence Alcohol outlet density was associated with sexual violence in several studies. No studies looked at the impacts of drinking environment policies (e.g., RSA) or sale-time polices on sexual violence perpetration
Palk et al. (2010)	Australia	Quantitative	Pre–Post-Design Evaluation	Policy	Sexual Offences	Compared incidents of sexual offenses before and after the implementation of a 3am lockout policy in nightclubs across one city	Sexual offenses requiring police attendance reduced by 33.7%
Powers and Leili (2018)	USA	Quantitative	Pre–Post-Design N = 155 venue staff 53% Male, age range 18–80 (*M* = 31.6) *Note 25% missing demographic data Evaluation	Bystander Intervention	Sexual Violence	Exploratory evaluation of the community based BarTAB bystander intervention training for venue staff. The program ran in two large cities in Florida	Overall, the training was effective at reducing rape myth acceptance (*p* < .01) and increasing bystanders’ willingness to intervene (*p* < .01). The training did not significantly decrease participants perceptions of barriers to intervening (*p* > .05)
Powers and Leili (2016)	USA	Qualitative	Focus Groups N = 39 venue staff 56.4% Females, aged 21–54 (*M* = 32.1, *SD* = 9.9)	General Discussion (role of venue staff in intervention, environmental interventions, barriers and facilitators of prevention)	Sexual Harassment and Assault	Participants were asked questions about what they thought ideal prevention programs and strategies should entail	Interventions used by staff included being an active bystander and making weaker drinks when male buys an intoxicated female a drink Suggestions for environmental strategies included adequate lighting, visible cameras, adequate staff and security numbers. Barriers to intervention were also discussed (e.g., taboo nature of sexual harm, ambiguous situations)
Prego-Meliro et al. (2022)	Spain	Qualitative	Theoretical Model	General Discussion (awareness-raising campaign)	Drug-facilitated Sexual Assault	Developing and applying a theoretical model to the prevention of drug-facilitated sexual assaults in nightlife venues	The authors discuss centering awareness-raising efforts on Routine Activity Theory. They specify that awareness messages should specifically target capable guardians, victims and perpetrators. Messages targeted at potential perpetrators could focus on modifying predatory behavior (e.g., targeting individuals who are too drunk to consent)
Quigg et al. (2020)	International	Mixed Methods	Scoping review	General discussion (literature review)	Sexual Violence	Broad review on nightlife-related sexual harm, with one component being prevention	Discussed 19 articles relating to awareness/media campaigns, bystander approaches, individual risk management and policy. Policy was the most common category of intervention followed by individual risk management. Only 2 studies had been evaluated
Quigg et al. (2021)	UK, Czech Republic, Portugal and Spain	Quantitative	Pre–Post-Design (surveys) N = 118 Staff 54.4% Male, aged 18—29 Evaluation	Bystander intervention (Awareness-Raising Component)	Sexual Violence	Evaluation of STOP-SV nightlife worker training across 3 pilot sites (Czech Republic, Portugal and Spain)	Post-training, participants were significantly more likely to be ready (*p* < .0001) and confident (*p* < .001) to intervene in sexual violence situations and less likely to agree with sexual violence myths (*p* < .01)
Quigg et al. (2022)	England	Quantitative	Pre–Post-Survey N = 206 venue workers, 60.6% Male, aged 22–29 Evaluation	Bystander Intervention	Sexual Violence	Evaluation of Good Night Out Campaign. Participants completed measures on sexual violence myth acceptance and readiness and confidence to intervene in sexual violence incidents	Participants were significantly less likely to agree with sexual violence myths post-training (p < .001). Compared to pre-training, participants were more likely to feel ready (p < .001) and confident to intervene (p < .001)
Toomey et al. (2012)	USA	Quantitative	Bayesian Hierarchical Inference Approach	Policy	Rape	Investigating the effect of alcohol establishment density on violent crime using crime data from the city’s police department	There was a significant, positive association between on-premise establishment density and rape (*p* < .05)
Wood and Shukla (2017)	England	Quantitative	Pre–Post-Design with Control Group N = 2045 Online-panel survey, Target audience before (n = 453) and after (n = 468) campaign launch Control group in area where campaign didn’t launch before (n = 628) and after (n = 496) Evaluation	Awareness-Raising Campaign	Unwanted Sexual Attention	Evaluation of “You wouldn’t sober, you shouldn’t drunk” campaign. Respondents were asked about campaign recall and subsequent changes in attitudes toward unwanted sexual attention	The experimental group with campaign recall had significantly lower tolerance of unwanted sexual attention compared with experimental group without campaign recall (*p* < .05) and control group (*p* < .005)
Wrightson-Hester and Allan (2022a)	Australia	Qualitative	Interviews N = 14 recent nightlife patrons, 50% Male, aged 19—29	General discussion (bystander intervention, barriers and facilitators of prevention)	Sexual Violence	Participants were presented with vignettes and asked open ended questions to facilitate discussions around barriers and facilitators of nightlife-related bystander interventions	Most participants would not intervene in a sexual violence situation. Barriers to intervening included ambiguity (e.g., not wanting to get involved where a relationship exists) and existing social norms (e.g., buttocks touching is common in nightlife venues) Participants would be more likely to intervene if the perpetrator’s behavior was persistent or intrusive or the recipient was their friend or a female

Sixteen records were identified in the gray literature search that discussed a specific intervention or prevention strategy. Most records were from high-income countries, including England (37.5%), the USA (25%), and Australia (25%). Only four (25%) of the 16 records contained outcome information. Table 3 characterizes the gray literature by intervention type and briefly describes the intervention. The majority of records described a bystander-related intervention (68.8%), followed by staff interventions (12.5%), police interventions (12.5%), and a venue safety charter (6.3%). Table 4 provides a summary of each record that was included in the review.

**Table 3:** Categorizing gray literature by intervention type

Intervention	Description	Title
Bystander-related interventions	i. Venue Staff Training ii. Bystander-specific awareness-raising campaign (targeted at nightlife patrons)	i. Good Night Out, Bristol Nights, Shout-Up!, Safe Bars, The Safe Bar Collective, Make Your Move, Project Night Light ii. Project Night Justice, #Safetosay, Stand up don’t stand by, Make Your Move, We Won’t Keep Quiet
Staff interventions	Interventions utilizing venue staff to prevent sexual harm	Ask for Angela (2 records)
Police interventions	Police officers targeting perpetrator behaviors	Project Vigilant, Operation Empower
Venue safety charter	Venues sign a charter whereby they must adhere to several pledges regarding the prevention and response of sexual harm	Women’s Night Safety Charter

**Table 4:** Summary of the gray literature records that were included in the current paper

Title, Author (Year)	Country	Intervention Type	Description	Findings (if applicable)
Project Night Justice. Crime Stoppers Victoria (2022)	Australia	Awareness-Raising Campaign (Bystander)	“Step up. Speak up.” campaign aimed to encourage bystanders to stop violence against women at night. Encourages reporting and venue training, through awareness raising (posters and website). If venues sign a charter they can access a free toolkit which provides practical guidelines around safety and reporting	N/A
Good Night Out (Australia). Full Stop Australia (2023)	Australia	Venue Staff Training and Accreditation	Educate venue staff on prevention and response to sexual harm. Three step accreditation process including the development of a venue agreement/policy, a two-hour staff training session (education and bystander de-escalation techniques), and communication (posters and certificate to display in venue)	N/A
#Safetosay. Walker et al. (2022)	Wales	Awareness-Raising Campaign (Bystander)	Evaluation report of the 4-week campaign (using data from survey of N = 265 respondents). The campaign encouraged patrons (aged 16–45) to speak up and respond to sexual harassment in nightlife venues. Materials were provided on social media, posters, a website (with bystander toolkit), bus linings and parking meter adverts	71% of respondents reported that the campaign helped them understand how to intervene safely in a sexual violence situation. Of the respondents who had seen the campaign (n = 50), 10% reported that the campaign had no effect on them
Bristol Nights (2021)	England	Venue Staff Training	Free anti-sexual harassment training offered online (self-guided or trainer facilitated) and in-person to all staff and performers who work in the night-time economy. The training encompasses education (e.g., relevant laws, acceptable and unacceptable behaviors), bystander intervention and best practice management of incidents of sexual harassment	N/A
Ask for Angela. Gloucestershire Rape and Sexual Abuse Centre (2021)	England	Venue Staff Intervention	Initiative that encourages women to discreetly ask venue staff for assistance (by asking for Angela) if they are in danger or are being sexually harassed. Stickers and posters displayed in female toilets explains the process to patrons. Staff will respond to the situation (e.g., evict perpetrator, call authorities)	N/A
Ask for Angela. South Australian government (2021)	Australia	Venue Staff Intervention	Initiative providing a discreet way for women to ask venue staff for assistance (by asking for Angela) if they are in danger or are being sexually harassed. The initiative aims to empower staff to address inappropriate sexual behavior. Venues received information sheets, posters and awareness training for staff. The posters were to be placed in discreet locations in the venue	N/A
Operation Empower. Bates et al. (2022)	England		Merseyside Police launched a proactive policing response in which they targeted individuals who displayed predatory behavior in and around nightlife settings. Officers undertook sexual violence bystander training. This initiative was evaluated using pre–post-training surveys, interviews/focus groups and nightlife-user surveys	52 (72.5% male) police officers completed the surveys. Post training, 71.2% of participants reported having a better understanding of how to respond to sexual violence. 90.4% of participants felt they had an increased knowledge of where to go for help in sexual violence cases. Officers reported environmental factors such as CCTV and lighting were beneficial in preventing and responding to sexual violence
Shout-Up! (2022)	England	Venue Staff Training and Accreditation	Educating venue staff on preventing and responding to sexual harm. Managers and supervisors completed an in-person bystander intervention training session and all other staff members complete an online training course. Venues must also create and implement a sexual-harassment policy	N/A
Stand Up, Stand up don’t stand by (2019)	America	Awareness-Raising (Bystander)	Website that provides tips on how to be an active bystander (e.g., keep an eye out for people who may need help; call back up—staff or security) and downloadable awareness-raising posters for venues. Also provides links to staff bystander training services in several cities	N/A
Safe Bars (2022)	America	Venue Staff Training	Training venue staff to stand up against sexual violence through education (e.g., prevalence of sexual violence in nightlife venues) and bystander strategies (e.g., how to respond safely). Also provide venues with Safe Bars materials to display to patrons	N/A
The Safe Bar Collective (2017)	America	Venue Staff Training	A 2-h training session aiming to educate venue staff about understanding and responding to sexual violence in nightlife settings. Venues are provided with a window decal to display Provided an internal evaluation of the first year of the program (note: no information about how survey was conducted)	Of the venue staff surveyed (N = 104), 88% of participants reported having a greater understanding of sexual violence, 90% of respondents reported that they were confident that they could implement the skills learned in the workshop
Project Vigilant. Magil et al. (2022)	England	Police Intervention	Covert officers trained in behavior observation, patrol designated areas (e.g., outside venues) on high-risk nights (normally weekends). The officers target perpetrator behavior. The covert officers then communicate with uniformed officers who then directly intervene	Initial internal evaluation results suggest that there was a 50% reduction in rape and 30% reduction in sexual assault Police officers commented that the project is giving officers more confidence in intervening in such behavior
Project Night Light. City of Adelaide (2023)	Australia	Venue Staff Training	A pilot program targeted at prioritizing women’s safety within nightlife venues. Twelve venues will participate in staff bystander training and two workers from each venue will be nominated as champions who will actively promote the safety of women	N/A
Make Your Move! Make You Move End Sexual Violence (2021)	America	Awareness-Raising Campaign (Bystander) Venue Staff Training and Accreditation	The poster campaign aimed to increase bystander intervention and understanding. Posters were displayed in bathroom stalls, advertisements were also placed in newspapers, social media and at the local theater (30 s advertisement shown before movie screenings) The venue staff training is a 2 and ½ hour workshop which focusses on recognizing and responding to sexually aggressive behavior. At least 70% of staff must be trained to receive certification	N/A
Women’s Night Safety Charter. Greater London Authority (2022)	England	Safety Charter	Nightlife venues can sign the charter and must abide by several pledges. The pledges include nominating an individual in the organization to promote women’s safety, encourage reporting, train staff in how to report and respond to incidents of sexual harm. Venues must also promote safety (e.g., through communications campaigns) and design spaces to make them safer for women	N/A
We Won’t Keep Quiet, Barcelona City Council (2018)	Spain	Awareness-Raising Campaign Venue-level Strategies	Barcelona City Council protocol for reducing sexual harm in private nightlife venues. Preventative actions suggested were not having discriminatory criteria for venue access, monitoring dark areas of venue, listening to all reports of sexual harm, not displaying promotional activities that incite discrimination against women. Detection and action strategies include staff training, having clear policies and steps to respond to incidents, assist victims and address the assailant We won’t keep quiet campaign—display campaign materials in venues and provide an informative leaflet to people who experience a serious assault	N/A

## Discussion

### Bystander Involvement, Interventions, and Campaigns

Bystander interventions have become increasingly popular in recent years and are theoretically based on work conducted by Latané and Darley (1970). They suggested that for bystanders to engage in helping behavior in a critical situation, they must move through five stages (notice the event, interpret it as an emergency, take personal responsibility to intervene, decide how to act, and then act; Latané & Darley, 1970). Routine activity theory posits that for a crime (i.e., sexual harm) to occur, there must be a motivated offender, an accessible target, and the absence of a capable guardian (Cohen & Felson, 1979). In theory, bystander intervention programs and campaigns should increase the likelihood of a capable guardian (i.e., an individual who has the skills to appropriately respond) being present, thereby reducing the likelihood of incidents of sexual harm escalating (Cook & Reynald, 2016). Bystander-related interventions covered the largest number of interventions identified. Currently, there is no consistent way in which these interventions have been categorized; therefore the current study has attempted to group them in a way that fits the emerging literature.

#### Venue Staff Bystander Interventions

From the peer-reviewed literature, five studies discussed venue staff bystander interventions (Graham et al., 2014; Hill & Megson, 2020; Powers & Leili, 2018; Quigg et al., 2021, 2022), with three of these studies being evaluations of staff bystander training (Powers & Leili, 2018; Quigg et al., 2021, 2022). All evaluations found that post-training, staff were significantly more ready and confident to intervene in incidents of sexual harm compared to pre-training. Less experienced staff obtained greater benefits from training (Powers & Leili, 2018), and staff’s rape myth acceptance significantly decreased post-training in the three evaluations. Knowledge around the ongoing effectiveness of staff bystander training is limited, with no studies investigating the long-term impacts of the training and only one study conducting a short-term follow-up (Quigg et al., 2021). It is important to assess the long-term impact of bystander programs, as factors such as high staff turnover may impact intervention effectiveness (Graham et al., 2004). Additionally, seven records from the gray literature search promoted similar venue staff training, with three of these records incorporating a venue accreditation component (Bristol Nights, 2021; City of Adelaide, 2023; Full Stop Australia, 2023; Make Your Move End Sexual Violence, 2021; Safe Bar Collective, 2017; Safe Bars, 2022; Shout-Up, 2022). While information was scarce for some records, all training entailed an active bystander section (e.g., how to respond safely) and an education section (e.g., understanding what constitutes sexual harm).

#### Patron Bystander Awareness Campaigns

Four bystander-specific awareness-raising campaigns targeted at nightlife patrons were identified in the gray literature search (Crime Stoppers Victoria, 2022; Make Your Move End Sexual Violence, 2021; Stand Up Don’t Stand By, 2019; Walker et al., 2022). The campaigns were advertised using a variety of methods, including social media, promotional websites, and posters, and provided suggestions about how to be an active bystander and intervene in a safe manner. An evaluative report on the #SafeToSay campaign in Wales indicated that 71% of respondents felt that their understanding of safe bystander responses had increased (Walker et al., 2022). However, the cross-sectional survey design could not establish whether there were tangible reductions in sexual harm due to the campaign. Two of the identified studies investigated the role of alcohol intoxication in patron bystanders (Ham et al., 2019, 2022). Ham et al. (2019) found that alcohol impacted the first two stages of the bystander intervention model (Latané & Darley, 1970), but not the final three stages. Therefore, intoxicated patrons may not reach the critical intervention point if they are impaired in the earlier stages (Ham et al., 2019).

#### Patron Bystander Approaches

Finally, nine articles discussed how patrons utilize active bystander approaches within nightlife venues. A common risk-management approach highlighted in in-depth interview (García-Carpintero et al., 2022), focus group (Brooks, 2011; Gunby et al., 2020), and online survey (Graham et al., 2017) research was the use of male friends and boyfriends to prevent or intervene in sexual harm incidents perpetrated by other males. This could be a problematic solution for male patrons, as research suggests that such involvement often leads to subsequent physical aggression (Kavanaugh & Anderson, 2009). Another similar theme among these studies and observational research was the use of peer-centered protective strategies to intervene in such incidents (e.g., put body between friend and potential perpetrator; Brooks, 2011; García-Carpintero et al., 2022; Gómez et al., 2022; Graham et al., 2014; Kavanaugh & Anderson, 2009).

#### Overview of Bystander Interventions

The growing interest in bystander-related interventions may be founded from social justice movements advocating for the responsibility of prevention to be taken away from victims (Williams et al., 2019). While evaluations of venue-staff bystander training suggest several positive effects, particularly for less experienced staff members, it is important to note that such interventions are reactionary rather than preventative. Bystander approaches do not cause reductions in future incidents of sexual harm; rather, they aim to stop them from escalating when they do occur. Additionally, the methodology utilized in existing literature (e.g., pre–post-survey design; Powers & Leili, 2018) does not allow us to determine whether patrons and staff are engaging in active bystander techniques in practice and, in turn, if this is leading to reductions in the incidence or severity of nightlife-related sexual harm.

While the review highlighted that nightlife patrons commonly report engaging in active bystander techniques, the extent to which venues and awareness campaigns should rely on patrons themselves to manage such incidents may be limited. Alcohol intoxication can impact patrons’ ability to become aware of dangerous situations and identify the need to intervene (Ham et al., 2019). This is consistent with in-depth interview and anonymous questionnaire research, which suggests that individual and situational factors can inhibit individuals’ ability to recognize incidents of sexual harm within nightlife and party contexts (Burn, 2009; Wrightson-Hester et al., 2022b). According to routine activity theory, bystander programs should decrease the risk of sexual harm escalating by increasing the likelihood of a capable guardian being present (Cook & Reynald, 2016). However, if patrons fail to identify incidents of sexual harm due to factors such as alcohol consumption, dim lighting, crowding, and noise level, this will likely limit their ability to act as a capable guardian and intervene.

### Policies, Laws, and Regulations

Eight articles discussed the impact of policies, laws, or regulations on the prevention of sexual harm (Benny et al., 2019; De Vocht et al., 2016; Hill & Megson, 2020; Hill et al., 2020; Khurana & Mahajan, 2022; Lippy & DeGue, 2016; Palk et al., 2010; Toomey et al., 2012). The majority of these articles were centered on alcohol and licensing policies at the district or state level. Duncan et al. (2022) conducted in-depth interviews with alcohol-related violence researchers. Some participants expressed concerns about alcohol policies unfairly impacting non-violent individuals, while others argued that the use of alcohol policies is justified given their efficacy in reducing women’s vulnerability to sexual violence (Duncan et al., 2022).

#### Alcohol Policies

In regard to specific alcohol policies, on-premise outlet density was positively associated with sexual violence (Lippy & DeGue, 2016; Toomey et al., 2012). Additionally, higher alcohol prices and taxes were associated with lower levels of sexual violence (Lippy & DeGue, 2016). A natural experiment found that sexual assaults decreased by at least 10% following a ban on the sale of hard liquor in bars in one state in India, using neighboring states as a control (Khurana & Mahajan, 2022). Similarly, Palk et al. (2010) found that sexual offenses requiring police attendance were reduced by approximately 34% following the implementation of a 3.a.m lockout policy in nightclubs across one Australian city using a pre–post-design. Finally, De Vocht et al. (2016) analyzed sexual crime rates in England using hierarchical growth modeling and found that sexual crime rates declined faster in areas with more stringent alcohol policies from 2009 to 2013; however, crime rates increased again following this period. This trend was found across a variety of alcohol-related crimes, with the authors providing no explanation for this change beyond stating that interpretation is difficult given various interacting factors. Collectively, these findings suggest that alcohol and licensing policies are an encouraging avenue for the prevention of sexual harm in nightlife settings. This is consistent with a wealth of literature that indicates that alcohol use is significantly associated with sexual harm perpetration and victimization (e.g., Abbey, 2002; Fung et al., 2021; Santos et al., 2015). As such, it follows that implementing widespread policies that limit or restrict the consumption of alcohol may lead to a decrease in incidents of nightlife-related sexual harm.

#### Venue-Level Policies

At the venue level, while no formal evaluations have been conducted, both researchers and patrons in the UK suggested that safe space policies were a strategy that may prevent sexual harm in nightlife spaces (Hill & Megson, 2020; Hill et al., 2020). These policies clearly outline to staff and patrons what is considered unacceptable behavior and explain how such behavior will be managed. Safe space policies were created by feminists during the Women’s Liberation Movement and are underpinned by values such as equality and respect (Hill et al., 2020; Keenan & Darms, 2013). While male managers in one study were concerned about such policies excluding males, the authors clarified that the main aim of these policies is not to exclude men but rather to advocate for and encourage safety among women and other groups (e.g., LGBTQIA+ ; Hill et al., 2020). This concept aligned with suggestions from Australian focus group participants, who discussed the need for venues to create and implement clear policies on responding to inappropriate patron behavior and reports of sexual harm (Fileborn, 2016).

### Awareness-Raising Campaigns

Six articles discussed awareness-raising campaigns as a preventative measure, with three articles describing or evaluating a specific campaign (Brooks, 2011; Carline et al., 2018; Gunby et al., 2017; Levine, 2018; Prego-Meliro et al., 2022; Wood & Shukla, 2017). A large-scale evaluation was conducted for the “You wouldn’t sober, you shouldn’t drunk” campaign in England, which was designed to influence existing social norms around unwanted sexual attention (Wood & Shukla, 2017). While the evaluation did not measure tangible reductions in unwanted sexual attention, the experimental group with campaign recall had a significantly lower tolerance of unwanted sexual attention than the experimental group without campaign recall or the control group. A further two focus-group studies discussed a novel campaign targeted at male nightlife patrons aged 18–24 years in England (Carline et al., 2018; Gunby et al., 2017). The campaign aimed to clarify the laws surrounding what constitutes rape. Very few participants were aware of the campaign and attributed its ineffectiveness to factors such as poor advertising material (e.g., model was not attractive and didn’t show enough skin) and their intoxication level while out (i.e., wouldn’t notice or take in the material; Gunby et al., 2017). The authors suggested that the campaign materials were in competition with more noticeable sexualized alcohol and venue advertisements (Gunby et al., 2017). In contrast, the campaign evaluated by Wood and Shukla (2017) was advertised through posters, social media, and cinema advertisements and was relatively successful in reducing the tolerance of unwanted sexual attention. While further investigation is needed, these initial findings suggest that campaign advertisement materials may be more noticeable, thereby more effective when advertised outside of the nightlife setting. Finally, one identified study suggested that awareness-raising campaigns should be grounded in routine activity theory, with specific messages targeted at capable guardians, potential victims, and perpetrators (Prego-Meliro et al., 2022). In order to address the scarcity of evidence in this area, future research should prioritize forming a theoretical basis for campaigns and conducting effective evaluations. Further, campaigns could be utilized for early intervention (e.g., within secondary schools) in order to address the wider social norms that underpin sexual harm.

### Police Interventions

Two policing interventions were identified in the gray literature search, which involved covert officers targeting perpetrator behavior in and around nightlife venues in England (Bates et al., 2022; Magill et al., 2022). In Operation Empower, officers undertook sexual violence bystander training, with the post-training survey evaluation finding that 71.2% of officers reported having a better understanding of how to respond to sexual violence (Bates et al., 2022). While the evaluation did not measure reductions in incidents of sexual harm, officers participating in focus groups reported feeling more confident following training, and nightlife patrons who were surveyed had positive opinions of the operation (Bates et al., 2022). An initial internal evaluation of Project Vigilant suggested that there was a 50% reduction in rape and a 30% reduction in sexual assault (Magill et al., 2022). No details were given on the nature of this evaluation or what methodology was used; therefore, these may not be generalizable to other precincts. Research suggests that there are greater reductions in crime in areas with greater policing resources (Machin & Marie, 2011); therefore, resource restraints across different jurisdictions may limit the feasibility and effectiveness of policing interventions. While the initial findings are promising, further investigation is required in order to determine the efficacy of police interventions.

### Implementation of Trained Care Worker Teams

One study utilized police recorded crime data to evaluate the effectiveness of a care worker group in the UK who were trained to protect the welfare of nightlife patrons and provide practical support to potential victims of sexual harm (“Drinkaware Crew”; Garius et al., 2020). The intervention did not significantly reduce police-reported sexual crimes following its implementation. The authors concluded that the data they utilized were not appropriate for the purpose of the evaluation. Research suggests that police data often underestimate the prevalence of alcohol-related violence in nightlife settings (Sutherland, 2002); therefore, the results may not be reflective of actual crime rates, supporting the authors conclusions about their data. As there is currently no conclusive evidence available, interventions involving trained care workers need to be further examined to determine their effectiveness in reducing sexual harm in nightlife venues.

### Staff Interventions

Two records discussed the staff intervention “Ask for Angela,” which is an initiative that encourages women to discreetly ask staff for help when they are at risk of being sexually harassed (Gloucestershire Rape & Sexual Abuse Centre, 2021; South Australian Government, 2021). No outcome information or evaluations of this intervention were identified in the search. This form of intervention relies on patrons themselves to recognize and report sexual harm, which, as mentioned above, can be problematic due to factors such as patron intoxication level (Ham et al., 2019). Moreover, venue staff who participated in focus group research in the USA reported that they would naturally keep an eye out on potentially harmful situations and coordinate with security staff when necessary (Powers & Leili, 2016). The venue staff in this study also suggested that having an adequate number of staff, security, and managers in venues could mitigate the risk of sexual harm. Patrons’ perceived effectiveness of security staff was mixed across studies (Anitha et al., 2021; Kavanaugh & Anderson, 2009). Kavanaugh and Anderson (2009) found that female patrons saw security as a useful resource for managing incidents of sexual harm. Whereas more recently, in-depth interview participants indicated that venue security often do not respond appropriately to reports of sexual harm, with participants suggesting that security staff often invalidate women’s experiences (Anitha et al., 2021).

Having adequate staffing is also important for reducing the risk of sexual harm directed at venue staff (Green, 2022). Interviews with venue workers in England found that newer staff often feel as though they must accept unwanted sexual attention and often need to be encouraged by more experienced staff to report incidents to management (Green, 2022). While interventions that aim to prevent sexual harm from occurring are ideal, reactive approaches (i.e., security staff intervening) to incidents of sexual harm are still necessary (Edwards et al., 2018). Creating clear policies and procedures for venue security to follow in response to witnessing or receiving reports of sexual harm may reduce incidents from escalating and lessen the tolerance of sexual harm within nightlife settings. These policies could also incorporate measures that address the risk of sexual harm to venue staff.

### Environmental Interventions

While no evaluations have been conducted, two studies discussed the potential of environmental interventions to reduce or prevent sexual harm (Forsyth, 2009; Powers & Leili, 2016). Observational research highlighted that music could influence the level of disorder and sexual activity among the crowd. Forsyth (2009) suggested that music can be used as a form of “soft policing” to control patrons. Sexual activity was most frequently reported in urban clubs that played R&B music (Forsyth, 2009), which may be explained by certain music genres facilitating an overt sexual atmosphere in the venue and resulting in greater incidents of unwanted sexual contact (Sanchez et al., 2019). Additionally, venue workers suggested that having adequate lighting and visible cameras in the venue would likely deter patrons from perpetrating sexual harm (Powers & Leili, 2016). These claims are supported by experienced police officers, who have reported that CCTV and good lighting are beneficial for sexual violence prevention and response in nightlife settings (Bates et al., 2022). Emerging research suggests that environmental characteristics such as lighting level, sexually violent advertising, reserved areas for sex, the presence of poles (i.e., for pole dancing), and venue type play a role in facilitating sexual behavior (Forsyth, 2009; Gunby et al., 2017; Sanchez et al., 2019; Wrightson-Hester et al., 2022b). Such venue-level attributes may encourage overtly sexual behavior among patrons and diminish their ability to distinguish between consensual and non-consensual conduct in this environment, thereby increasing the likelihood of sexual harm occurring (Wrightson-Hester et al., 2022b). Given the evolving research on the relationship between sexual harm and environmental factors, environmental interventions are a promising avenue for future research. Preventative measures that target environmental components of nightlife settings (e.g., lighting level) may be an effective way to shift the responsibility of preventing sexual harm away from patrons themselves.

### Barriers and Facilitators of Sexual Harm Intervention

A number of articles discussed the barriers and facilitators of implementing sexual harm prevention strategies at both individual and venue levels (Hill & Megson, 2020; Levine, 2018; Powers & Leili, 2016; Wrightson-Hester et al., 2022a). For venues, the main barriers noted were financial or resource constraints (e.g., cannot afford staff training) and building researcher-venue relationships (Hill & Megson, 2020; Levine, 2018). Further, bar staff in one study questioned whether management would adopt preventative measures (e.g., staff training) as their practices are generally centered on making a profit (Powers & Leili, 2016). Previous research suggests that high staff turnover is also a notable barrier faced by venues in the implementation of interventions (Graham et al., 2004). Facilitators of change identified at the venue level included networking between venues and having female venue owners and promoters (Hill & Megson, 2020). At an individual level, consistent barriers for intervening in potential sexual harm situations for both venue staff and patrons included existing social norms (e.g., sexual harm is normalized within these settings) and ambiguous situations (e.g., unable to determine if they are in a relationship or if the exchange is consensual; Powers & Leili, 2016; Wrightson-Hester et al., 2022a). Finally, patrons may be more likely to intervene in a potentially harmful situation if the victim is female and a friend or if the perpetrator’s behavior was malicious or premeditated (Wrightson-Hester et al., 2022a).

### Strengths and Limitations

The findings of the current study should be considered in light of its limitations. Firstly, as only English-language articles were included, the review may have excluded pertinent research. Similarly, as the majority of the identified articles represent interventions being discussed and trialed in high-income countries, the findings might not be generalizable to nightlife settings in low-income countries. Further, due to the broad nature of sexual harm, not all evaluative studies measured comparable outcome variables, which may have led to differences across studies. This review was not able to determine the most effective intervention; however, by including articles that were not evaluations, a more comprehensive understanding of potential nightlife-related interventions was developed. Despite these limitations, this review had a number of strengths, including the inclusion of gray literature, which likely reduces publication bias. Further, existing research largely ignores theoretical justifications for nightlife-related sexual harm and its prevention. The current review included theoretical discussion in an attempt to shift toward a prevention approach that incorporates both empirical evidence and theory.

### Conclusion

In order to inform the development and implementation of evidence-based interventions, a comprehensive understanding of the nature and effectiveness of existing nightlife-related sexual harm prevention strategies is essential. Existing literature is predominantly based on qualitative data and discussions around potentially effective strategies. The findings from the current study indicate that while research in the area is increasing, high-quality evaluative studies remain limited. Promising avenues for intervention that were identified were targeted alcohol regulations and venue policies (e.g., safe space policies). Existing prevention efforts have a strong focus on placing the responsibility of prevention on to the patrons themselves (Quigg et al., 2020). Given the increasing evidence indicating that environmental factors are associated with sexual harm in nightlife venues, future research should consider the development of environmental interventions. The area cannot progress without the development of interventions based on sound theoretical work and empirical evidence and a substantial shift toward more rigorous evaluative practices. In order to see widespread uptake of preventative practices, researchers and key stakeholders should consider the barriers faced by venues when designing interventions. It is also important to address the barriers to prevention posed by venues, such as the hyper-sexualized environment and overt objectification of women and other marginalized groups (e.g., through sexually violent advertising; Sanchez et al., 2019).

## Data Availability

Not applicable.

## Appendix

**Table:**

Topic area	No.	Search terms	No.	Exclusion terms
Terms related to sexual harm	1	Sexual n3 harm	41	Child
	2	Sexual n3 aggres*	42	Sex worker
	3	Sexual n3 harass*	43	Sport#
	4	Sexual n3 assaul*	44	Nightmare#
	5	Sexual n3 offenc*	45	Plant#
	6	Sexual n3 violen*	46	Magnetic
	7	Sexual n3 misconduct	47	OR/ 41–46
	8	Sexual n3 crime		
	9	Unwanted n3 sexual		
	10	Rape		
	11	Coerc*		
	12	Non#consensual		
	13	OR/ 1–12		
Terms related to nightlife	14	Night*		
	15	Entertain*		
	16	Venue#		
	17	Club#		
	18	Pub#		
	19	Bar#		
	20	Restaurant#		
	21	Patron#		
	22	OR/ 14–21		
Terms related to interventions	23	Interve*		
	24	Preven*		
	25	Regulat*		
	26	Restrict*		
	27	Policy		
	28	Policies		
	29	Ban#		
	30	Legislat*		
	31	Measure#		
	32	Reduc*		
	33	Bystander#		
	34	Awareness		
	35	Strateg*		
	36	Safety		
	37	Campaig*		
	38	Tactic#		
	39	OR/23–38		
Combine	40	13 AND 22 AND 39	48	40 NOT 47
